# PTENP1/miR-20a/PTEN axis contributes to breast cancer progression by regulating PTEN via PI3K/AKT pathway

**DOI:** 10.1186/s13046-019-1260-6

**Published:** 2019-06-13

**Authors:** Xue Gao, Tao Qin, Jun Mao, Jun Zhang, Shujun Fan, Ying Lu, Zhigang Sun, Qingqing Zhang, Bo Song, Lianhong Li

**Affiliations:** 1 Department of Pathology, Dalian Medical University, 9 Lushunnan Road Xiduan, Dalian, 116044 Liaoning Province China; 2 Department of Pathology, the First Hospital of Dalian Medical University, Dalian, 116027 Liaoning Province China; 3 Key Laboratory of Tumor Stem Cell Research of Liaoning Province, Dalian Medical University, Dalian, 116044 Liaoning Province China; 4 Teaching Laboratory of Morphology, Dalian Medical University, Dalian, 116044 Liaoning Province China; 5 Teaching Affairs Department, Dalian Medical University, Dalian, 116044 Liaoning Province China

**Keywords:** Breast cancer, PTENP1, miR-20a, PTEN, PI3K/AKT pathway

## Abstract

**Background:**

Long non-coding RNA PTENP1, the pseudogene of PTEN tumor suppressor, has been reported to exert its tumor suppressive function via modulation of PTEN expression in many malignancies, including breast cancer (BC). However, whether the PTENP1/miR-20a/PTEN axis exists and how it functions in BC progression remains elusive.

**Methods:**

The levels of PTENP1, PTEN and miR-20a were measured by qRT-PCR. Furthermore, the breast cancer cells proliferation was further measured by CCK8 assay, colony formation assays, EDU and Ki67 staining. The migratory and invasive ability was determined by transwell assay. Flow cytometry, JC-1 and TUNEL assays were conducted to show the occurrence of apoptosis. Xenograft model was used to show the tumorigenesis of breast cancer cells.

**Results:**

We analyzed PTENP1 and PTEN levels in clinical BC samples and cell lines, and found that PTENP1 and PTEN were confirmed and closely correlated with the malignancy of BC cell lines and poor clinical prognosis. Moreover, alteration of PTENP1 affects BC cell proliferation, invasion, tumorigenesis and chemoresistance to adriamycin (ADR). Bioinformatic analysis and dual-luciferase reporter gene assay predicted that PTENP1 was a direct target of miR-20a, which was clarified an alternative effect on BC aggressiveness phenotype. In addition, PTENP1 functioned as an endogenous sponge of miR-20a to regulate PTEN expression, which mediated BC cells proliferation, invasion and drug resistance via activation the phosphatidylinositol-3 kinase (PI3K)/AKT pathway. PI3K inhibitor LY294002 or siAkt also prevented BC cells progression.

**Conclusion:**

Collectively, these data indicated that PTENP1/miR-20a/PTEN axis involved in the malignant behaviors of BC cells, illuminating the possible mechanism mediated by PTEN via PI3K/Akt pathway. Targeting PTENP1/miR-20a/PTEN may provide a potential diagnosis and treatment strategy for BC.

**Electronic supplementary material:**

The online version of this article (10.1186/s13046-019-1260-6) contains supplementary material, which is available to authorized users.

## Background

Breast cancer (BC) is the most common cause of cancer mortality among women, accounting for 16% of cancer deaths in adult women [1]. Despite improvements in the prognosis of BC seen in recent decades, additional therapeutic advances are needed, particularly for patients with metastatic/advanced disease [2]. Furthermore, the drug resistance in the course of chemotherapy has brought great threat to breast cancer patients [3], especially as chemoresistance limits the effectiveness of chemotherapeutic agents to a large extent. Therefore, it is imperative to clarify the underlying molecular mechanism during BC progression.

Long non-coding RNAs (LncRNAs) are defined as a class of non-protein coding transcripts over 200 nucleotides, and are now emerging as crucial regulators of cellular processes and diseases, and their aberrant transcription can lead to altered expression of target genes involved in cancer pathways and functions [4]. By binding to the associated gene of cancer, lncRNAs function as oncogenes or tumor suppressors in human cancer. For example, lncRNA PTENP1, the pseudogene of PTEN tumor suppressor, contains a highly homologous region upstream of the 3′UTR of PTEN, can regulate PTEN expression, thus exerts an effect on the process of carcinogenesis [5]. PTENP1 also participates in repressing cell proliferation, inhibiting migration, and promoting apoptosis [6, 7]. Recent studies have shown that lncRNAs, as competing endogenous RNAs (ceRNA), play important roles in modulating miRNA function through binding sites [8]. Enhanced PTENP1 could inhibit BC cell growth, metastasis and tumourigenicity by inhibiting miR-19b and facilitating PTEN in BC [6]. Consequently, miRNAs further affect the downstream protein-coding genes by binding to its 3′-UTR. Wei et al. showed that miR-130a as an oncogenic miRNA that targets PTEN to drive malignant cell survival and tumor growth [9]. MiR-20a acts as a negative regulator of PTEN, and mediates the proliferation, migration and apoptosis of multiple myeloma [10]. Similarly, we have found that PTENP1 influences the biological function of miR-20a in BC progression.

The PI3K/Akt pathway is activated subsequent to RTK activation. Hyperactivation of PI3K/Akt signaling has been reported in many types of human cancers, thus targeting the regulators in this pathway has attractive therapeutic potential [11]. Ectopic expression of PTENP1 resultes in the upregulation of PTEN, accompanies by the blockage of PI3K/Akt pathway and growth inhibition in prostate and renal cancer cells [12, 13]. MiR-106b and miR-93 regulate BC cell migration, invasion and proliferation by suppression of PTEN via PI3K/Akt pathway, which could be blocked by upregulation of PTEN [14]. MiR-130b targets PTEN to reduce drug resistance, proliferation and apoptosis of BC cells via the PI3K/Akt pathway [15]. However, PTENP1 and miR-20a affect PTEN, an important process in BC progression, is not clear.

In the present study, the association of downregulated PTENP1 and PTEN and BC progression was examined. LncRNA PTENP1 was evaluated as a molecular sponge for miR-20a, and these ncRNAs further regulated PTEN. Relative function mechanism assays revealed that PTENP1/miR-20a/PTEN axis exerted its function in BC partly through PI3K/Akt signaling pathway.

## Materials and methods

### Samples from BC patients

A total of 52 previously diagnostic BC patients who received surgical operation at the First Affiliated Hospital of Dalian Medical University from April 2014 to January 2018 were enrolled in this study. The study and its informed consent have been examined and certified by the Ethics Committee of the First Affiliated Hospital of Dalian Medical University (YJ-KY-FB-2017-32). In accordance with the International Union against Cancer (UICC), the samples were identified BC tissues and their adjacent noncancerous tissues. The samples were maintained in liquid nitrogen for later experiments.

### Cell culture

The human BC cell lines MDA-MB-231, T-47D and MCF-7 were obtained from KeygenBiotech Co. Ltd. (Nanjing, China). The mammary epithelium MCF-10A was purchased from ATCC cell banking. The BC cells were cultured in DMEM, supplied with 10% fetal bovine serum (Gibco, Grand Island, NY, USA) and 1% penicillin-streptomycin (Gibco, Grand Island, NY, USA) at 37 °C in a humidified and 5% CO_2_ incubator. MCF-10A cells were maintained in DMEM-F12 media supplemented with hydrocortisone (0.5 μg/ml), insulin (10 μg/ml), hEGF (20 ng/ml) and 10% (v/v) FBS. Adriamycin (Sigma, St Louis, MO, USA) was added to parental cell cultures in stepwise increasing concentrations from 1 mg/l to 5 mg/l for 2 months to develop an adriamycin-resistant (ADR) subline, named MCF-7/ADR and T47D/ADR, correspondingly. To maintain the resistant phenotype, MCF-7/ADR and T47D/ADR cells were kept in the medium containing 1 mg/l adriamycin (ADR) and were cultured in drug-free medium for 48 h before the experiments.

### Real-time PCR analysis

Total RNA was isolated from tissues and BC cell lines by RNeasy Mini Kit (Qiagen, Valencia, CA). RNA was reverse transcribed to cDNA using Reverse Transcription Kit (Invitrogen, CA, USA). The qRT-PCR was performed under an ABI Prism7500 fast real-time PCR system (Applied Biosystems, Foster City, CA) with mixing a QuantiTect SYBR Green PCR Kit (Qiagen, Valencia, CA). Relative RNA expression was calculated by ΔΔCt method with normalization to U6 small nuclear RNA.

### Western blot analysis

Total cell lysates were prepared with RIPA lysis buffer (KeyGEN, Nanjing, China). The proteins were separated by 10% SDS-PAGE gels and then transferred to PVDF membranes (Millipore, Bedford, MA, USA). TBST with 5% skim milk powder was used for blocking the PVDF membranes. Then, the blots were incubated with different primary antibodies at 4 °C overnight. After incubating with secondary antibodies, the bands were detected by an ECL Western blot kit (Thermo Fisher Scientific, USA) and analyzed by LabWorks (TM ver4.6, UVP, BioImaging Systems, NY, USA). GAPDH was used as control.

### Cell transfection and RNA interference

PCR production of PTEN ampliation was cloned into pmirGLO vector (Promega). MiR-20a mimic, inhibitor and miR-NC were synthesized by GenePharma Co.Ltd. (Suzhou, China). LncRNA PTENP1 pcDNA3.1 vector (PTENP1, Invitrogen, CA, USA), LV-NC, LV-PTENP1, siPTENP1, shPTENP1, siAkt, siSCR and shSCR were obtained from GenePharma Co.Ltd. (Suzhou, China). The transfection assay was conducted with Lipofectamine 2000 (Invitrogen, Carlsbad, CA, USA) for incubation. The transfected efficiency was measured by qRT-PCR.

### Dual luciferase reporter gene assay

A pmirGLO Dual-Luciferase miRNA Target Expression Vector was purchased from GenePharma Co.Ltd. (Suzhou, China). Firefly luciferase functioned as primary reporter to regulate mRNA expression, and renilla luciferase was used as a normalized control. Co-transfection was conducted with lipofectamine 2000 for 48 h, the dual luciferase reporter assay system (Promega) was utilized according to the manufacturer’s instruction. The relative luciferase activity was calculated as the ratio of frefly luciferase activity versus renilla luciferase activity. Data were shown as the mean value ± SD and each experiment was performed thrice.

### CCK8 assay

Cell proliferative ability was investigated by using cell counting kit-8 (CCK-8; Dojindo, Japan). Cells (1 × 10^3^ per well) were plated into 96-well plate with the corresponding medium, and cultured in a humidified incubator at 37 °C. 11 μLCCK8 were added into the plate for 4 h. The spectrometric absorbance was measured by microplate reader (Model 680; Bio-199 Rad, Hercules, CA, USA) at 490 nm.

The drug resistance to ADR was detected by CCK-8. Different concentration of ADR was added into 96-well plate after seeding the cells. Similarly, the absorbance was then measured to evaluate the drug resistance to ADR of BC cells. Each experiment was performed thrice.

### Focus formation assay

Single-cell suspension was obtained and then seeded in 6-well plate with 1 × 10^3^ per well. The medium was changed every 4 days. 12 days later, the foci were formed obviously. The colonies were fixed by 4% paraformaldehyde for 20 min, and then stained with 0.2% crystal violet. The colonies were photographed and counted.

### Cell invasion assay

Cell invasive ability was measured using ECMatrix gel (Chemicon)-coated transwell inserts, respectively (Trevigen, City of Gaithersburg, Maryland, USA). 5 × 10^4^ cells were harvested in serum-free DMEM and added to the upper chamber. Medium containing 10% FCS was added to the bottom chamber, and cells were allowed to invade for 24 h at 37 °C. The upper side cells were removed by a cotton swab. The invading cells were counted to estimate the invasive capacity. Five random fields were analyzed for each chamber.

### Flow cytometry analysis

Cells were incubated with different concentration of ADR for 48 h. Annexin-V-FITC apoptosis detection kit (BD, Franklin Lakes, NJ, USA) was used to measure cell apoptosis. 2 × 10^3^ cells were harvested and adjusted in 100 μL binding buffer. Annexin V and propidium iodide were used to stain for 10 min avoiding lights, and 400 μL binding buffer was added into the cell suspension. The apoptosis cells were detected by FACS Calibur (Becton-Dickinson, CA, USA).

### Immunofluorescence staining

BC cells were added in culture dishes and fixed with 4% paraformaldehyde for 20 min. BC cells were treated with 0.2% Triton X-100 for 3 min, and incubated with 5% BSA for 1 h. The primary antibody was added into the dish overnight at 4 °C. Slides were then washed three times with PBS and incubated 1 h with secondary antibody. The cells were stained by 4, 6-diamino-2-phenylindole (DAPI, Sigma-Aldrich, St Louis, MO, USA) in PBS for nuclear staining. Images were taken in a Carl Zeiss fluorescent microscope (Carl Zeiss Microscopy).

### TUNEL assay (terminal deoxynucleotidyl transferase dUTP nick end labelling)

TUNEL assay was carried out to measure the fragmented DNA of apoptotic cells. Apoptotic cells were induced by ADR, and fixed by 4% formaldehyde for 25 min at 4 °C. The cells were permeabilized by 0.2% TritonX-100 for 5 min. Then the cells were equilibrated with 100 μL Equilibration buffer for 10 min at room temperature. Cellswere labeled with 50 μL TdT reaction mix at 37 °C for 1 h. SSC buffer was used to stop the reaction and stained nuclei with DAPI. The images were obtained by fluorescence microscopy.

### RNA immunoprecipitation (RIP) assay

The Magna RIPTM RNA Binding Protein Immunoprecipitation Kit (Millipore, USA) was used to conduct RNA immunoprecipitation (RIP) assay. The endogenous miR-20a which combined with PTENP1 was pulled down. The cell lysis were collected and incubated in RIP buffer containing magnetic bead conjugated to either a human anti-Ago2 antibody (Millipore) or negative control IgG and was used to precipitate the cell extracts. The expression levels of PTENP1 and miR-20a in the precipitates were analyzed by qPCR.

### In vivo antitumor activity

4-week-old female athymic nude mice were purchased from the Model Animal Research Institute of Nanjing University. Approximately, 1 × 10^7^ cells were injected subcutaneously into the right flank of each nude mouse, respectively. In addition, to evaluate the chemosensitivity effect of PTENP1, the treatment groups received 7 mg/kg ADR i.p. three times a week for 3 weeks. The mice were humanely killed and their tumors were photographed. The tumor volume was calculated. These experiments were approved by the Committee on the Ethics of Animal Experiments of the Dalian Medical University, China.

### Immunohistochemistry (IHC) staining

Human BC samples and xenograft tumors were collected and performed on paraffin-embedded sections. 4 μm-thick sections were deparaffinized, rehydrated and then immersed with 3% hydrogen peroxide for 10 min to quench endogenous peroxidase and labeled with antibodies at 4 °C overnight. The slides were stained with the secondary streptavidin-horseradish peroxidase-conjugated antibody (Santa Cruz Biotech, Santa Cruz, CA) for 1 h. The slides were then counterstained with hematoxylin for 30s and cover slipped.

### Statistical analysis

Data were expressed as means ± standard deviation (SD). SPSS 17.0 software was used to analyze the experimental data. Student’s t-test was performed to compare two different groups. The one-way analysis of variance (ANOVA) was used to determine the significant difference of multiple groups. The survival curves were calculated by Kaplan-Meier method, and the difference was assessed by a log-rank test. Spearman’s correlation analysis was used to identify the association between miRNAs and mRNA expression. *P* < 0.05 was considered statistically signifcant.

## Results

### PTENP1 and PTEN are concomitantly downregulated in BC tissues and cell lines

**Fig. 1:**
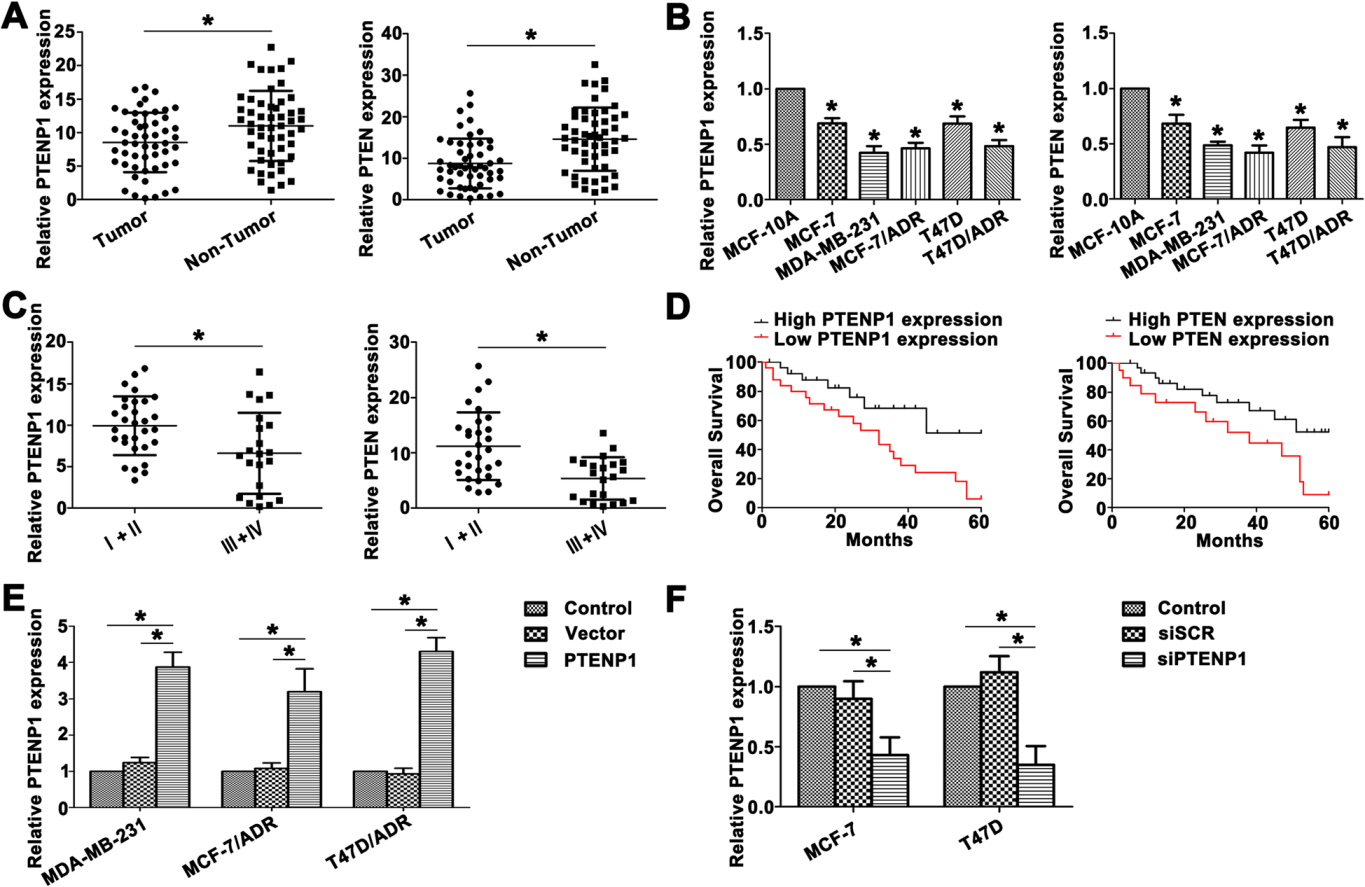
PTENP1 and PTEN are concomitantly downregulated in BC tissues and cell lines. **a** Higher PTENP1 was observed in the nontumor tissues than BC tissues. PTEN was downregulated in BC tissues. **b** The levels of PTENP1 and PTEN were identified in MCF-10A and BC cell lines. **c** Relative PTENP1 and PTEN levels showed lower tendency in advanced stages. **d** The overall survival curves (OS) of PTENP1 and PTEN were presented. **e** PTENP1 expression was extremely higher with transfection of PTENP1 compared to the control groups. **f** Decreased PTENP1 level was detected in the transfected MCF-7 and T47D cells. Data are the means ± SD of triplicate determinants (*P < 0.05)

Researchers have shown PTENP1 to be lost or downregulated in various cancers. To evaluate PTENP1 status in BC, we examined PTENP1 expression between 52 pairs of BC tissues and the corresponding adjacent tissues from the same patients. PTENP1 showed a lower level in tumor tissues relative to the paired adjacent tissues (Fig. 1a). The expression of PTEN, the parental gene of PTENP1, in the same paired biopsies was reduced in tumor tissues (Fig. 1a). In addition, the identical condition was also observed in the cell level of BC (Fig. 1b). Down expression of PTENP1 and PTEN was confirmed in MDA-MB-231 and ADR resistant BC cell MCF7/ADR and T47D/ADR.

We next examined the significance of PTENP1 and PTEN expression correlated to the pathological features of BC patients. PTENP1 and PTEN levels were closely correlated with advanced BC stages, and BC patients on stage III and IV exhibited lower PTENP1 and PTEN levels (Fig. 1c). Kaplan-Meier analysis was used to analyze the correlation between PTENP1 or PTEN expression and the overall survival rate. As shown in Fig. 1d, the PTENP1 or PTEN downregulated patients showed signifcant poor prognosis.

To better understand the role of PTENP1 in BC progression, we manipulated the expression of PTENP1 by transfecting PTENP1 or siPTENP1 in BC cell lines. As shown in Fig. 1e and f, PTENP1 was significantly upregulated in transfected MDA-MB-231, MCF7/ADR and T47D/ADR cells, and siPTENP1 effectively inhibited the endogenous PTENP1 level in MCF-7 and T47D cells. In addition, the length of PTENP1 and the non-coding potential also identified through online bioinformatic tools (Additional file 1: Figure S1). These data indicated that low levels of PTENP1 and PTEN might promote the BC progression and associate with the poor clinical prognosis.

### Overexpression of PTENP1 suppresses BC progression

**Fig. 2:**
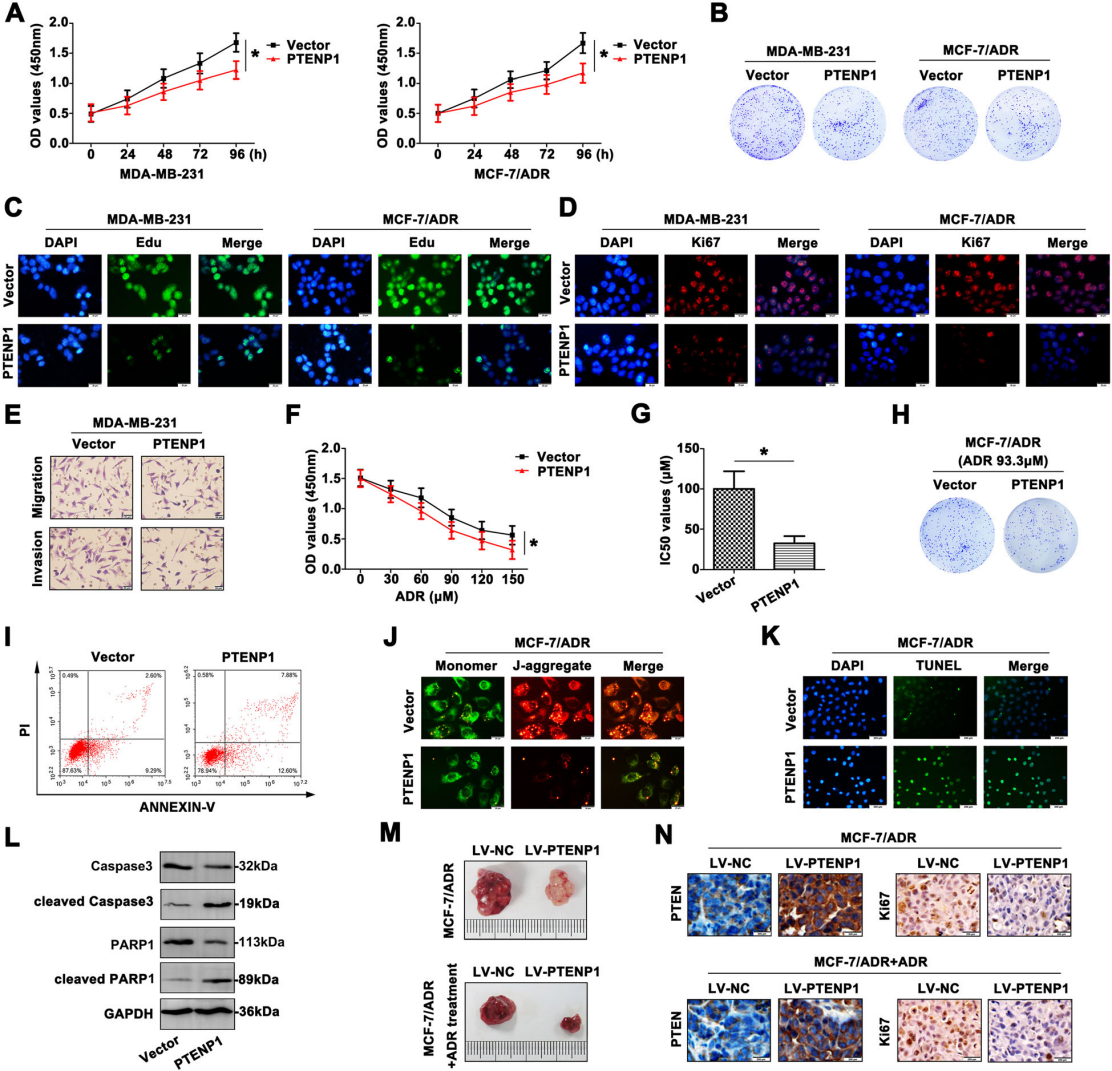
Overexpression of PTENP1 suppresses BC progression. **a** CCK8 assays were conducted to detect the growth of MDA-MB-231 and MCF-7/ADR cells. **b** The inhibitory proliferative effect of PTENP1 on MDA-MB-231 and MCF-7/ADR cells was determined by colony formation assay. **c** The proliferation of BC cells was measured by Edu staining. Green fluorescence: Edu, blue fluorescence: DAPI (Scale bar = 20 μm). **d** Ki67 staining also showed the repressed proliferation of PTENP1 transfected BC cells. Red fluorescence: Ki67, blue fluorescence: DAPI (Scale bar = 20 μm). **e** The migration and invasion of transfected MDA-MB-231 cells were suppressed by transwell experiment (Scale bar = 20 μm). **f** The chemoresistance to ADR of MCF-7/ADR cells was assessed by CCK8 assays. **g** The IC_50_ value was computed in the transfected MCF-7/ADR cells. **h** With ADR treatment, the colony formation of PTENP1 transfected MCF-7/ADR cells was more inhibited. **i** With ADR treatment, the apoptotic rate was determined by flow cytometry. **j** The mitochondrial membrane potential was measured by JC-1 staining. Green fluorescence: the monomer, red fluorescence: the J-aggregates, orange fluorescence: merged photo (Scale bar = 20 μm). **k** The occurrence of apoptosis was further confirmed by TUNEL assay (Scale bar = 200 μm). **l** The expression of caspase-related apoptotic molecules was determined by western blot. **m** The tumorigenesis was detected by xenograft model. The nude mice treated with PTENP1 revealed smaller tumor volume compared to the control group. The tumor was further inhibited in response to ADR treatment. **n** The levels of PTEN and Ki67 were determined by IHC staining. Data are the means ± SD of triplicate determinants (*P < 0.05), (Scale bar = 200 μm)

In order to explore the functional signifcance of PTENP1 upregulation on BC cell progression, CCK8 assays were used to identify the growth rate of BC cells. The results indicated that overexpression of PTENP1 attenuated BC cell viability (Fig. 2a). The colony formation assay also proved a decreased tendency after BC cells transfected with PTENP1 (Fig. 2b). Moreover, Edu showed a remarkable function in evaluating cell proliferation, which usually was involved in DNA synthesis. The merged images were presented, and showed that overexpression of PTENP1 reduced BC cells proliferation (Fig. 2c). In accordance with Edu staining, Ki67 expressed a weaken fluorescence intensity in BC cells transfected with PTENP1 (Fig. 2d), indicating that PTENP1 overexpression could inhibit BC cell growth. As shown in Fig. 2e, high PTENP1 level impeded the migratory and invasive capability of MDA-MB-231 cell compared to the cell transfected with vector, further demonstrating a tumor suppressive role of PTENP1 in BC cells.

Following PTENP1 overexpression, the reversal chemoresistance caused by PTENP1 was performed. Interestingly, when PTENP1 overexpression cells were incubated with the presence of the chemotherapeutic agent ADR, the cells demonstrated a reduced capability to proliferate compared with their control groups (Fig. 2f). The IC_50_ values were significantly decreased in PTENP1 overexpression groups compared with the controls (Fig. 2g). In response to ADR treatment, overexpressed PTENP1 attenuated the resistant cell colony formation (Fig. 2h), suggesting that PTENP1 overexpression in combination with chemotherapy might be a potentially viable treatment strategy. Moreover, upregulation of PTENP1 significantly enhanced the ability of chemotherapy-induced apoptosis in BC cell lines (Fig. 2i). The collapse of mitochondrial tansmembrane potential showed the occurrence of apoptosis. Mitochondrial membrane potential damage was a key event during the occurrence of apoptosis, which was caused by the activation of caspases, and cytochrome c released into cytosol. JC-1 content was detected by immunofluorescence staining, indicating the apoptosis caused by overexpressed PTENP1 (Fig. 2j). High fluorescence intensity was captured during the apoptosis occurred after overexpressing PTENP1 by fluorescence microscope (Fig. 2k). Apoptosis was also assessed by the appearance of caspase-3 cleavage after western blot. As shown in Fig. 2l, with drug treatment, BC cell lines transfected with PTENP1 expressed increased levels of cleaved caspase3 and cleaved PARP1, and decreased caspase3 and PARP1.

To further assess the chemosensitivity to ADR in vivo, mouse xenograft studies were performed. Overexpressed PTENP1 significantly inhibited tumor growth. In a further study in the ADR treatment PTENP1 overexpression model, the primary tumor volume was found to decrease with ADR treatment but overall the decrease was at a significantly faster rate than the untreated group (Fig. 2m), suggesting the effect of PTENP1-mediated function concomitant application of ADR in vivo*.* As shown in Fig. 2n, the expression of PTEN and Ki67 in xenograft tumor was also verified by IHC staining. Thus, overexpression of PTENP1 modulated BC cell proliferation, metastasis, apoptosis and tumorigenicity, as well as exhibited more sensitive to ADR.

### Low PTENP1 level enhances the malignant behavior of BC cells

**Fig. 3:**
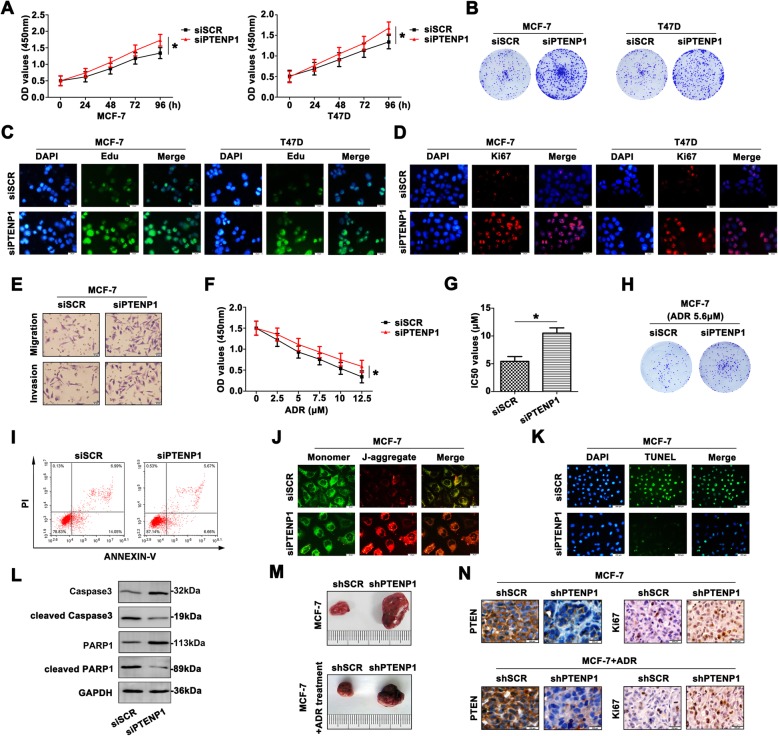
Low PTENP1 level enhances the malignant behavior of BC cells. **a** The viability of transfected BC cells were detected by CCK8 assays at 0, 24, 48,72, 96 h. **b** Knockdown of PTENP1 enhanced the colony formation in BC cells. **c** The proliferation of siPTENP1 transfected cells was increased by Edu staining (Scale bar = 20 μm). **d** Ki67 staining also showed intensive proliferation (Scale bar = 20 μm). **e** The aggressiveness was enhanced with knocking down PTENP1 in MCF-7 cells (Scale bar = 20 μm). **f** The siPTENP1-MCF-7 cells revealed more resistance to ADR. **g** Higher IC_50_ value was also proved the enhanced chemoresistance to ADR. **h** Weakened colony formation ability was shown in response to ADR. **i** More resistance to ADR was shown in siPTENP1-MCF-7 cells. Low apoptosis rate was detected by flow cytometry. **j** JC-1 staining assay showed altered mitochondrial membrane potential with siPTENP1 transfection. Green fluorescence: the monomer, red fluorescence: the J-aggregates, orange fluorescence: merged photo (Scale bar = 20 μm). **k** TUNEL assay confirmed the incidence of apoptosis (Scale bar = 200 μm). **l** Apoptosis-related molecules expression was determined by western blot. **m** The xenografted tumors were presented with or without ADR treatment. **n** PTEN and Ki67 levels were determined by IHC staining. Data are the means ± SD of triplicate determinants (*P < 0.05) (Scale bar = 200 μm)

To decipher the biological function of PTENP1 by forcing its expression in MCF-7 and T47D cells, downexpression of PTENP1 led to an increase in cell growth (Fig. 3a). In accordance with the findings of the proliferation assay, the colony numbers of siPTENP1 cells were remarkably increased (Fig. 3b). To intuitively observe the proliferation in BC cells with low PTENP1 expression, Edu staining (Fig. 3c) and Ki67 (Fig. 3d) staining were carried out. As shown in Fig. 3e, MCF-7 transfected with siPTENP1 obtained a more aggressive characteristic than the cells transfected with siSCR. For this part, we identified the inhibitory role of PTENP1 in BC malignancy.

Furthermore, down-expression of PTENP1 promoted MCF-7 and T47D cell chemoresistance to ADR (Fig. 3f). IC_50_ values were significantly higher in the siPTENP1 transfected cells than the control group (Fig. 3g). Colony formation assay further proved MCF-7 and T47D cell lines had a variable degree in response to chemotherapeutic treatment (Fig. 3h). In addition, the ADR significantly induced apoptosis in the both cell lines (Fig. 3i). JC-1 staining also showed an increased J-aggregates in CRC cells transfected with siPTENP1 (Fig. 3j). Moreover, TUNEL assay showed less occurrence of apoptosis after transfecting siPTENP1 (Fig. 3k). Altered level of full length caspase3, PARP1, cleaved caspase3 and cleaved PARP1 were also detected by western blot, suggesting that apoptosis indeed occurred (Fig. 3l).

Next, the antitumor activity of ADR against PTENP1-driven tumor growth in nude mice was also assessed. ADR treatment significantly reduced MCF-7 tumor growth (Fig. 3m). IHC staining was performed on tumors to indicate the expression of PTEN and Ki67 in tumors (Fig. 3n). Therefore, downregulation of PTENP1 promoted BC cell proliferation, metastasis and cell survival in response to ADR.

### PTENP1 is a direct target of miR-20a and a positive regulator of PTEN

**Fig. 4:**
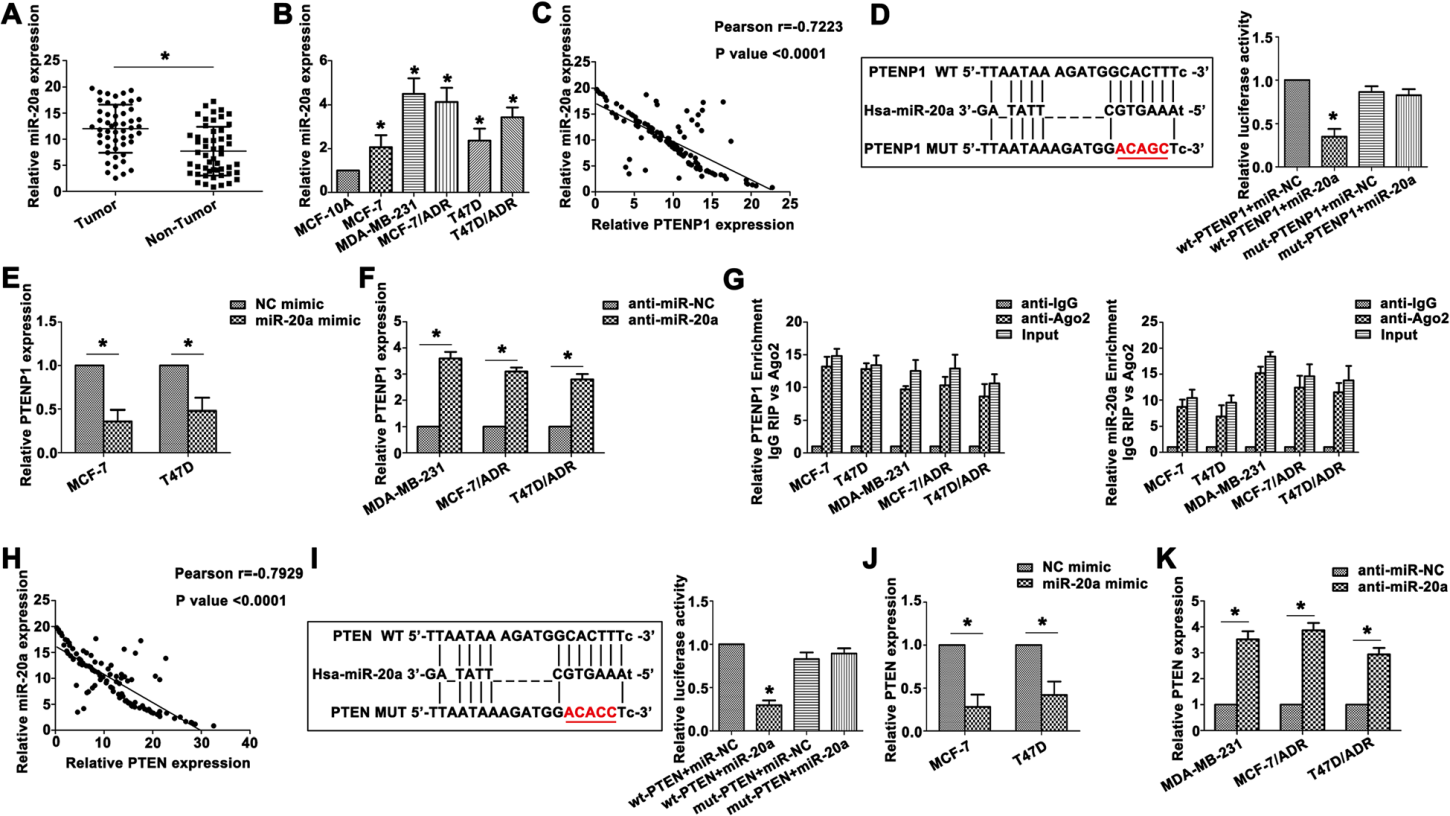
PTENP1 is a direct target of miR-26a and a positive regulator of PTEN. **a** Relative miR-20a expression was identified by qRT-PCR between BC tumor tissues and corresponding nontumor tissues. **b** Relative miR-20a expression was detected in MCF-10A and BC cell lines. **c** The negative correlation between PTENP1 and miR-20a was analyzed. **d** The predicted sequence aligment was shown, and dual-luciferase reporter assay confirmed the direct binding between PTENP1 and miR-20a. **e** Overexpressed miR-20a enhanced PTENP1 level in transfected MCF-7 and T47D cells. **f** MiR-20a inhibitor promoted PTENP1 expression in transfected MDA-MB-231, MCF-7/ADR and T47D/ADR cells. **g** The co-precipitated RNA was identified by RNA immunoprecipitation experiment. PTENP1 and miR-20a were shown as fold enrichment in Ago2. **h** The negative correlation was confirmed by Spearman’s correlation analysis. **i** The predicted binding sites were presented. The directed binding was confirmed by dual-luciferase reporter assay. **j** Downregulation of PTEN was detected in miR-20a mimic transfected MCF-7 and T47D cells. **k** Inhibition of miR-20a increased PTEN expression in MDA-MB-231, MCF-7/ADR and T47D/ADR cells. Data are the means ± SD of triplicate determinants (*P < 0.05)

Recently, ceRNA have generated substantial interest and have been reported in many cancers. Bioinformatic analysis predicts that miR-20a is closely associated with PTENP1. Interestingly, miR-20a showed certain difference between BC tissues and the adjacent tissues (Fig. 4a). Furthermore, MDA-MB-231 and ADR resistant BC cells revealed increased miR-20a level (Fig. 4b), indicating that miR-20a was frequently up-regulated in BC. A significant negative correlation was observed between PTENP1 and miR-20a expression (Fig. 4c). According to the bioinformatic analysis, we determined the predicted binding sites. Dual-luciferase reporter gene assay confirmed that PTENP1 was a direct target of miR-20a (Fig. 4d). MiR-20a mimic decreased the PTENP1 level in MCF-7 and T47D cells (Fig. 4e). In contrast, anti-miR-20a dramatically up-regulated PTENP1 expression in MDA-MB-231, MCF-7/ADR and T47D/ADR cells (Fig. 4f), indicating miR-20a could be a negative regulator of PTENP1. To verify whether PTENP1 associates with miRNP, RIP assay was performed on BC cell line extracts using anti-Ago2 antibody. PTENP1 and miR-20a were significantly enriched in Ago2-containing immunoprecipitate compared with control immunoglobulin G (IgG) immunoprecipitate (Fig. 4g), providing further evidence to the association of PTENP1 and miR-20a.

Next, pearson correlation coefficient analysis showed a significant negative correlation between miR-20a and PTEN in BC patients (Fig. 4h). We also performed dual-luciferase assay to confirm the interaction between miR-20a and PTEN (Fig. 4i), confirming that PTEN was a direct target of miR-20a. MiR-20a mimic significantly decreased expression of PTEN in MCF-7 and T47D cells (Fig. 4j). MiR-20a inhibitor also increased PTEN expression (Fig. 4k), indicating that there was a strong inverse correlation between expression of PTEN and miR-20a. PTENP1 might act as a tumor suppressor by endogenously competing with miR-20a, recovering the suppressed function of PTEN in BC.

### MiR-20a reverses the tumor suppressive function of PTENP1 by regulating PTEN expression in BC progression

**Fig. 5:**
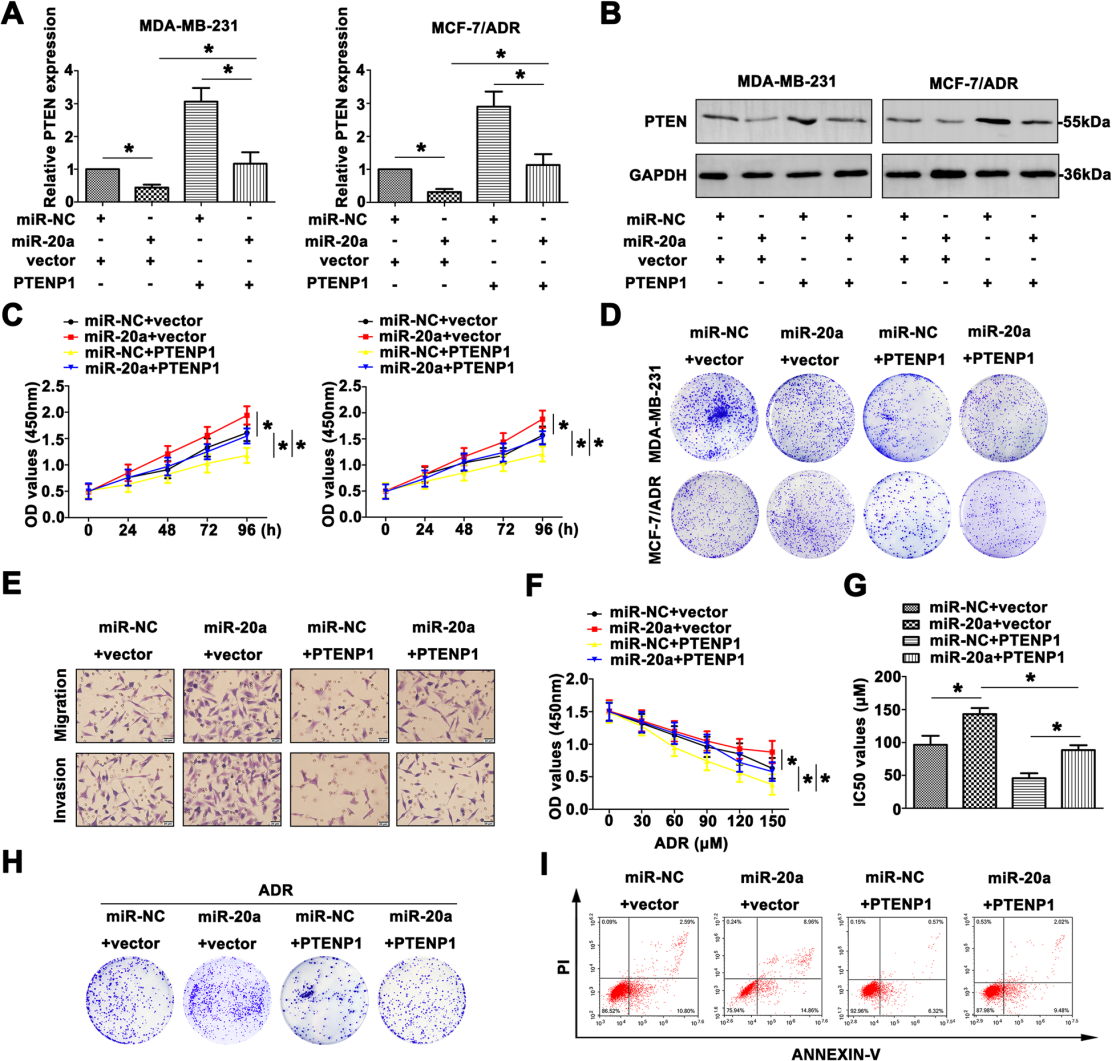
MiR-20a reverses the tumor suppressive function of PTENP1 by regulating PTEN expression in BC progression. **a** Relative PTEN expression was measured by qRT-PCR in the cells transfected with miR-20a mimic or PTENP1. **b** PTEN protein level was detected by western blot. **c** The variety of growth rates were confirmed by CCK8 assay. **d** Colony formation assay was conducted to show the proliferative formation with different treated BC cell lines. **e** The altered migration and invasion were detected by transwell assay (Scale bar = 20 μm). **f** The chemoresistance to ADR was identified by CCK8 assay. **g** The IC_50_ values of different groups were calculated. **h** The colony formation was shown in MCF-7/ADR cells in response to ADR. **i** The altered apoptotic rate was detected by flow cytometry. Data are means ± SD of three independent assays (*P < 0.05)

To investigate the relationships among PTENP1, miR-20a and PTEN and their effects on the BC development, we overexpressed PTENP1 and miR-20a in MDA-MB-231 and MCF-7/ADR cells. The mRNA and protein levels of PTEN were down-regulated by miR-20a mimic in these cells, whereas PTENP1 overexpression enhanced PTEN level. Co-transfection of miR-20a and PTENP1 showed that miR-20a partially abrogated the increase in PTEN level by PTENP1, suggesting that PTENP1 regulated the miR-20a target gene PTEN (Fig. 5a, b). Cell viability was significantly decreased in BC cells transfected with PTENP1, whereas the viability was reversed with co-transfection with miR-20a (Fig. 5c). Colony formation assay also depicted low numbers in BC cells transfected with PTENP1, and the colony formation ability was regained with transfected miR-20a mimic (Fig. 5d). As shown in Fig. 5e, in comparison to the control, MDA-MB-231 transfected with PTENP1 exhibited low aggressiveness, which was increased in MDA-MB-231 transfected with miR-20a. However, co-transfected PTENP1 and miR-20a depicted a revised migration and invasion than the cell only transfected with PTENP1.

To determine whether up-regulated PTENP1 and miR-20a were critical for MCF-7/ADR growth following ADR treatment, drug susceptibility assay was performed. As demonstrated in Fig. 5f, miR-20a mimic promoted MCF-7/ADR cell growth after ADR treatment. Interestingly, up-regulated PTENP1 inhibited the growth of MCF-7/ADR cells, and this growth could be reversed by miR-20a mimic. Furthermore, the IC_50_ of ADR on MCF-7/ADR cells was detected (Fig. 5g). With ADR treatment, the colony formation number was counted (Fig. 5h). Finally, cell apoptosis induced by PTENP1 or miR-20a in MCF-7/ADR cells after ADR treatment was detected by FCM (Fig. 5i). In short, all the outcomes above explained the function and regulatory mechanism of PTENP1/miR-20a/PTEN axis in the BC development.

### Inhibition of miR-20a reverses the promotional effect of siPTENP1 by mediating PTEN expression in BC progression

**Fig. 6:**
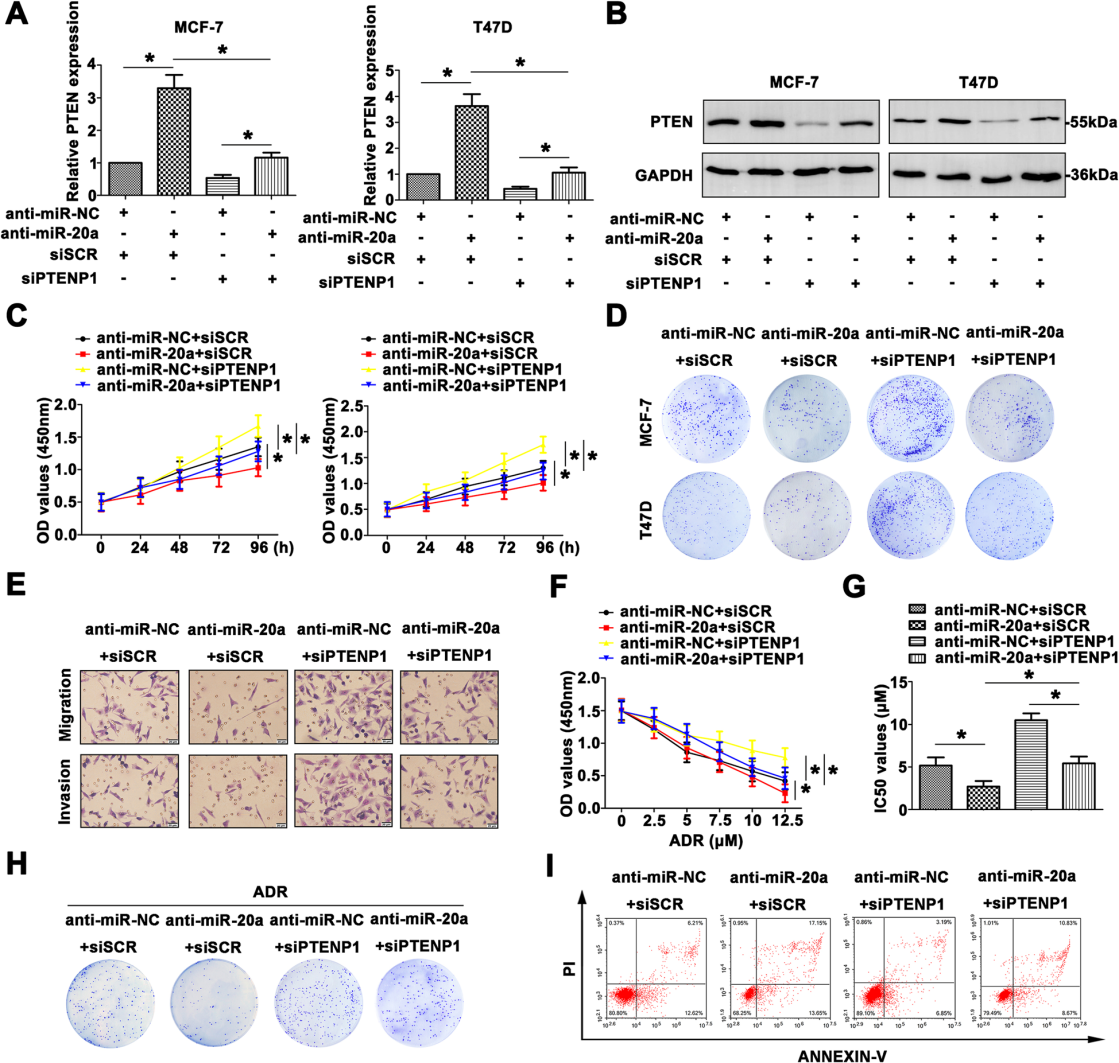
Inhibition of miR-20a reverses the promotional effect of siPTENP1 by mediating PTEN expression in BC progression. **a** PTEN mRNA expression was identified with the treatment of miR-20a inhibitor or siPTENP1. **b** PTEN protein level was detected by western blot. **c** The proliferation was measured by CCK8 assays. **d** Colony formation assay was used to measure the colony formation of transfected cells. **e** The aggressiveness was determined by transwell assay (Scale bar = 20 μm). **f** CCK8 assays were carried out to assess the chemoresistance to ADR with different treated BC cells. **g** IC_50_ values were calculated in differential treated MCF-7 cells. **h** In response to ADR, the colony formation was measured in transfected MCF-7 cells. **i** The AnnexinV and PI staining was used to determine the occurrence of apoptosis. Data are means ± SD of three independent assays (*P < 0.05)

To further determine the role of PTENP1-miR-20a-PTEN axis in BC progression, we down-regulated the expression of PTENP1 and miR-20a in MCF-7 and T47D cells. Increased PTEN was detected in the cells transfected with miR-20a inhibitor, and knockdown of PTENP1 decreased PTEN expression in BC cell lines (Fig. 6a, b). Co-transfection of anti-miR-20a and siPTENP1 showed that anti-miR-20a partially restored the suppression of PTEN level by siPTENP1. The growth rate was much higher in the cells transfected with siPTENP1, and lower in the cells transfected with miR-26a inhibitor than the control groups. Co-transfection of anti-miR-20a and siPTENP1showed that anti-miR-20a suppressed the proliferation promoted by siPTENP1 (Fig. 6c). Colony formation assay also depicted the same tendency (Fig. 6d). The migratory and invasive abilities were also reconsolidated by transfection with anti-miR-20a, siPTENP1 or co-transfection (Fig. 6e).

Furthermore, the potential function of PTENP1-miR-20a-PTEN pathway to ADR resistance was evaluated. The MCF-7 cells became sensitive to ADR after anti-miR-20a, while cells transfected with siPTENP1 remained resistant to ADR. Anti-miR-20a attenuated the cell sensibility to ADR induced by siPTENP1 (Fig. 6f). The IC_50_ values showed similar tendency (Fig. 6g). Colony formation assay further proved MCF-7 cells had a variable degree in response to ADR treatment (Fig. 6h). As shown Fig. 6i, treatment with ADR, anti-miR-20a reduced cell apoptosis, whereas siPTENP1 promoted apoptosis. Co-transfection of anti-miR-20a and siPTENP1 showed that anti-miR-20a restored apoptosis promoted by siPTENP1. These data suggest that miR-20a reverses the antitumor effect of siPTENP1 by regulating PTEN in BC progression.

### PTENP1/miR-20a/PTEN axis activates PI3K/Akt pathway in PI3K/Akt- mediated BC cell progression

**Fig. 7:**
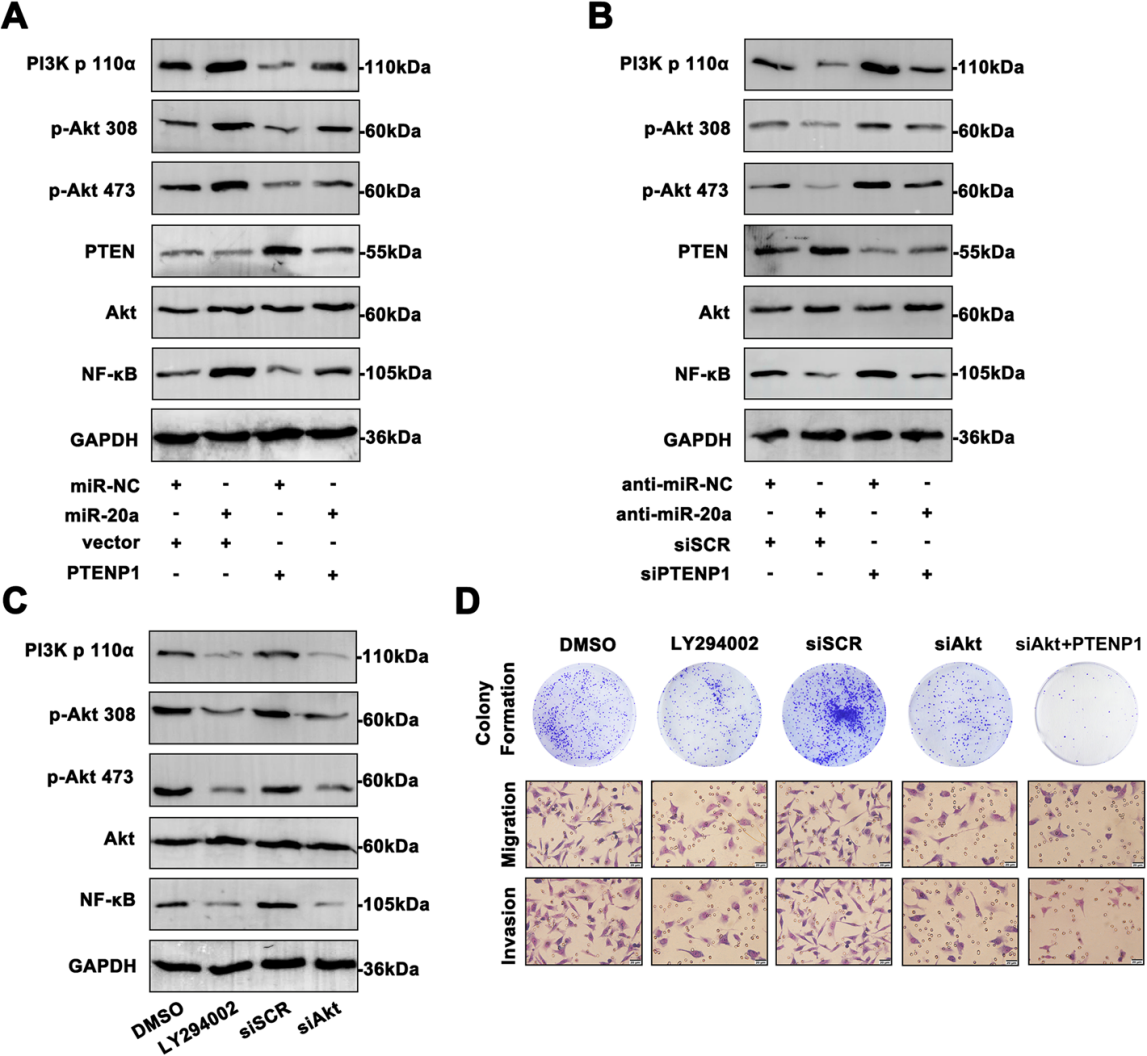
PTENP1/miR-20a/PTEN axis activates PI3K/Akt pathway in PI3K/AKT- mediated BC cell progression. **a** The main molecules of PI3K/Akt pathway were determined by western blot after transfection with miR-20a mimic or PTENP1. **b** The main molecules of PI3K/Akt pathway were measured with treatment of miR-20a inhibitor or siPTENP1. **c** The PI3K/Akt pathway was repressed by LY294002 and Akt siRNA. **d** Using colony formation assay and transwell assay, the attenuated proliferation and aggressiveness were shown in the cells transfected with LY294002, Akt siRNA or combination of siAkt and PTENP1. Data are means ± SD of three independent assays (*P < 0.05) (Scale bar = 20 μm)

Next, to investigate whether the PTENP1/miR-20a/PTEN axis could regulate PI3K/Akt signaling, we examined the effect of PTENP1/miR-20a/PTEN axis on the phosphorylation levels of PI3K/Akt pathway in BC cells. As shown in Fig. 7a, overexpressed miR-20a promoted PI3K/Akt signal activity in MDA-MB-231 cells. Upregulation of PTENP1 attenuated the pathway activation. However, co-transfection of miR-20a and PTENP1 in MDA-MB-231 cells significantly reversed the activity of pathway in relation to the cells transfected with miR-20a or PTENP1. Similarly, co-transfection of inmiR-20a and siPTENP1 in HCT-8/5-FU cells revealed a reversal effect on the phosphorylated status in the cells transfected inmiR-20a or siPTENP1 alone (Fig. 7b). Moreover, regulation of miR-20a and PTENP1 affected PTEN level, exhibiting regulatory role of PTENP1/miR-20a/PTEN axis in PI3K/Akt signaling.

To further understand the significance of PI3K/Akt pathway in BC progression, MDA-MB-231 cells were treated with PI3K inhibitor (LY294002) or siAkt. Treatment with LY294002 or siAkt significantly inhibited the phosphorylation of PI3K/Akt pathway in MDA-MB-231 cells (Fig. 7c). Accordingly, the proliferative rates and invasive ability were decreased in MDA-MB-231 cells treated with LY294002 or siAkt than the control cells (Fig. 7d). In addition, combination of siAkt and PTENP1 showed decreased cell proliferation and aggressiveness. Moreover, siAkt induced highly apoptotic cell rates compared to the siSCR group, demonstrating the potential utilization of PTENP1 for clinical BC targeting therapy (Additional file 2: Figure S2). These results suggested that PTENP1/miR-20a/PTEN axis could mediate the metastatic ability of cancer cells possibly by affecting PI3K/Akt activation.

## Discussion

Metastasis and chemoresistance lead to the treatment failure of BC patients. Interestingly, lncRNAs are reported to be critical regulators involved in tumour-related progression. Thus, we investigated the ceRNA-dependent role of the lncRNA PTENP1 in the development of BC. This study provided us the first clarification into the potential mechanism that PTENP1-miR-20a-PTEN network modulated the BC progression via PI3K/AKT pathway.

Recently, lncRNA PTENP1 and PTEN expression were found to be decreased in some cancer types, including hepatocellular carcinoma (HCC) [16], gastric cancer [7] and head and neck squamous cell carcinoma (HNSCC) [17]. We assessed PTENP1 and PTEN expression in BC tissues and cell lines, and the results showed down-regulation in BC tissues compared with adjacent normal tissues. In line with the results of our study, Li et al. reported decreased PTENP1 expression in BC cells, indicating such a decrease in expression may be important in oncogenesis [6]. Furthermore, our study also found low expression of PTENP1 and PTEN to be closely related to advanced TNM stage and overall survival in BC. Low levels of PTENP1 have been correlated with worse overall survival and disease-free survival rates of HNSCC patients [17], consistent with our results. Dysregulation of lncRNAs often led to the tumorigenesis and the malignant progression. PTENP1 over-expression resulted in the growth inhibition of cancer cells both in vitro and in vivo, suggesting that PTENP1 played a tumor suppressive role in HCC [16]. In esophageal squamous cell carcinoma (ESCC), overexpression of PTENP1 resulted in inhibited proliferation [18]. In the present study, we found that ectopic expression of PTENP1 led to inhibition of the tumor growth, colony formation, invasion and xenograft tumor growth of BC. Our results indicated that PTENP1 might function as potential therapy target of BC.

Recent evidence has suggested that competitive endogenous RNAs (ceRNAs) are important regulatory molecules in cancer and their dysregulation may contribute to cancer pathogenesis. For example, enhanced PTENP1 could inhibit BC cell growth, metastasis and tumourigenicity by inhibiting miR-19b and facilitating PTEN in BC [6]. MiR-19b and miR-20a were members of crucial oncogene miR-17-92 clusters. Although similar oncogenetic effects of miR-20a and 19b were existed on tumor procession, the overall BC cell malignancy should be better verified. In gastric cancer, PTENP1 was confirmed by binding miR-106b/ miR-93, in a ceRNA modulation manner, further affected PTEN level [7]. PTENP1 acted as a competing endogenous RNA to protect PTEN transcripts from being inhibited by miR-21, and consequently inhibited proliferation and colony formation in oral squamous cell carcinoma (OSCC) [19]. MiRNAs also play an important role in the ceRNA network through combining with target mRNA, inhibiting the action of mRNA expression [20]. MiR-20a has been shown to be aberrantly expressed in BC and regulated cancer aggressiveness by target genes. MiR-20a-5p was highly expressed in both triple-negative breast cancer (TNBC) tissues and cell lines, and promoted the growth of TNBC cells through targeting Runt-related transcription factor 3 (RUNX3) [21]. High mobility group AT-hook 2 (HMGA2) was a target of miR-20a-5p, which significantly induced carcinogenesis of BC [22]. Thus, the molecular mechanism was further clarified that PTENP1 acted as a ceRNA of miR-20a in BC progression. In the ceRNA network constructed in this paper, we found that PTENP1 expression was inversely correlated to miR-20a level in BC cell lines and patients. MiR-20a bound to PTENP1 in a sequence-specific manner and regulated PTENP1 expression. On the other hand, miR-20a was negatively correlated with PTEN level. Moreover, PTEN was a direct target of miR-20a and could be regulated by either miR-20a overexpression or inhibition. Additionally, altered level of PTENP1 and miR-20a was significantly associated with PTEN expression, and impacted BC malignancy. These results provide additional evidence to the reciprocal repression loop of PTENP1/miR-20a/PTEN in a functional aspect of BC development.

PI3K is responsible for coordinating a diverse range of cellular functions, including proliferation, cell survival, degradation and cell migration [23]. As a key oncogentic signaling pathway, PI3K/Akt pathway plays a pivotal role in the development of many cancers, including BC [24]. Additionally, NF-κB showed critical modulation on tumor cells malignancy and progression by regulating numerous genes transcriptionally. Although the involvement of PI3K/Akt pathway in BC has been declared, PTENP1-miR-20a-PTEN network mediated the signaling cascade has not been fully explained so far. In this present study, we found that PTEN level was regulated by PTENP1 and miR-20a, whereas PTEN participated in suppressing the proliferation of cancer cells via negatively regulating the PI3K/Akt pathway [25, 26], which suggested that PTEN was important in malignant transformation of cancer cells. In agreement with these observations, this study showed that altered PTENP1 and miR-20a could effectively influence the expression of PI3K/Akt pathway molecules via PTEN levels. On the other hand, treatment with LY294002 or siAkt signifcantly inhibited the phosphorylation of PI3K/Akt. As expected, the cell colony formation and invasion were attenuated in BC cells treated with LY294002 or siAkt. It was reasonable to conclude that the regulatory effects of PTENP1/miR-20a/PTEN crosstalk on cell aggressiveness could at least be partially mediated via PI3K/Akt pathway.

## Conclusion

In conclusion, PTENP1, a tumor suppressor, was low in BC tissues and cell lines, and has the potential to be a prognostic biomarker of BC. Moreover, PTENP1 was discovered to act as a sponge for miR-20a, regulating the downstream PTEN/PI3K/Akt pathway. PTENP1 mediated cell proliferation, metastasis, apoptosis and chemoresistance by endogenously competing with miR-20a through PTEN via PI3K/Akt pathway in BC. The results suggest that PTENP1 may be used as a novel target for clinical diagnosis and therapeutic application of BC.

## Additional files

Additional file 1: **Figure S1.** Detailed information of PTENP1. (A) The predicted PTENP1 length was around 3.9kb by lncrnadb. (B) PTENP1 was predicted as non-coding RNA by ORF Finder and PhyloCSF analysis. (JPG 787 kb)

Additional file 2: **Figure S2.** SiAkt treatment induced highly apoptotic cell rates compared with the siSCR group. (JPG 265 kb)

## Data Availability

Source data and reagents are available from the corresponding author upon reasonable request.

## Abbreviations

3’UTR: 3′-untranslated region
BC: Breast cancer
FCM: Flow cytometry
LncRNAs: Long non-coding RNAs
miRNAs: Micro RNAs
OS: Overall survival
