# Correction: PTENP1/miR-20a/PTEN axis contributes to breast cancer progression by regulating PTEN via PI3K/AKT pathway

**DOI:** 10.1186/s13046-023-02783-1

**Published:** 2023-08-09

**Authors:** Xue Gao, Tao Qin, Jun Mao, Jun Zhang, Shujun Fan, Ying Lu, Zhigang Sun, Qingqing Zhang, Bo Song, Lianhong Li

**Affiliations:** 1 Department of Pathology, Dalian Medical University, 9 Lushunnan Road Xiduan, Dalian, 116044 Liaoning Province China; 2 Department of Pathology, the First Hospital of Dalian Medical University, Dalian, 116027 Liaoning Province China; 3 Key Laboratory of Tumor Stem Cell Research of Liaoning Province, Dalian Medical University, Dalian, 116044 Liaoning Province China; 4 Teaching Laboratory of Morphology, Dalian Medical University, Dalian, 116044 Liaoning Province China; 5 Teaching Affairs Department, Dalian Medical University, Dalian, 116044 Liaoning Province China

**Correction:** ***J Exp Clin Cancer Res*** **38, 256 (2019)**

**https://doi.org/10.1186/s13046-019-1260-6**

Following publication of the original article [1], the authors identified an errors in the images of Figs. 3 and 6, specifically:

- Fig. 3E – Migration, group of siSCR
- Fig. 6D – T47D, group of anti-miR-NC + siPTENP1
- Fig. 6E – Migration, group of anti-miR-20a + siSCR

The correct figures are given below.

**Fig. 3:**
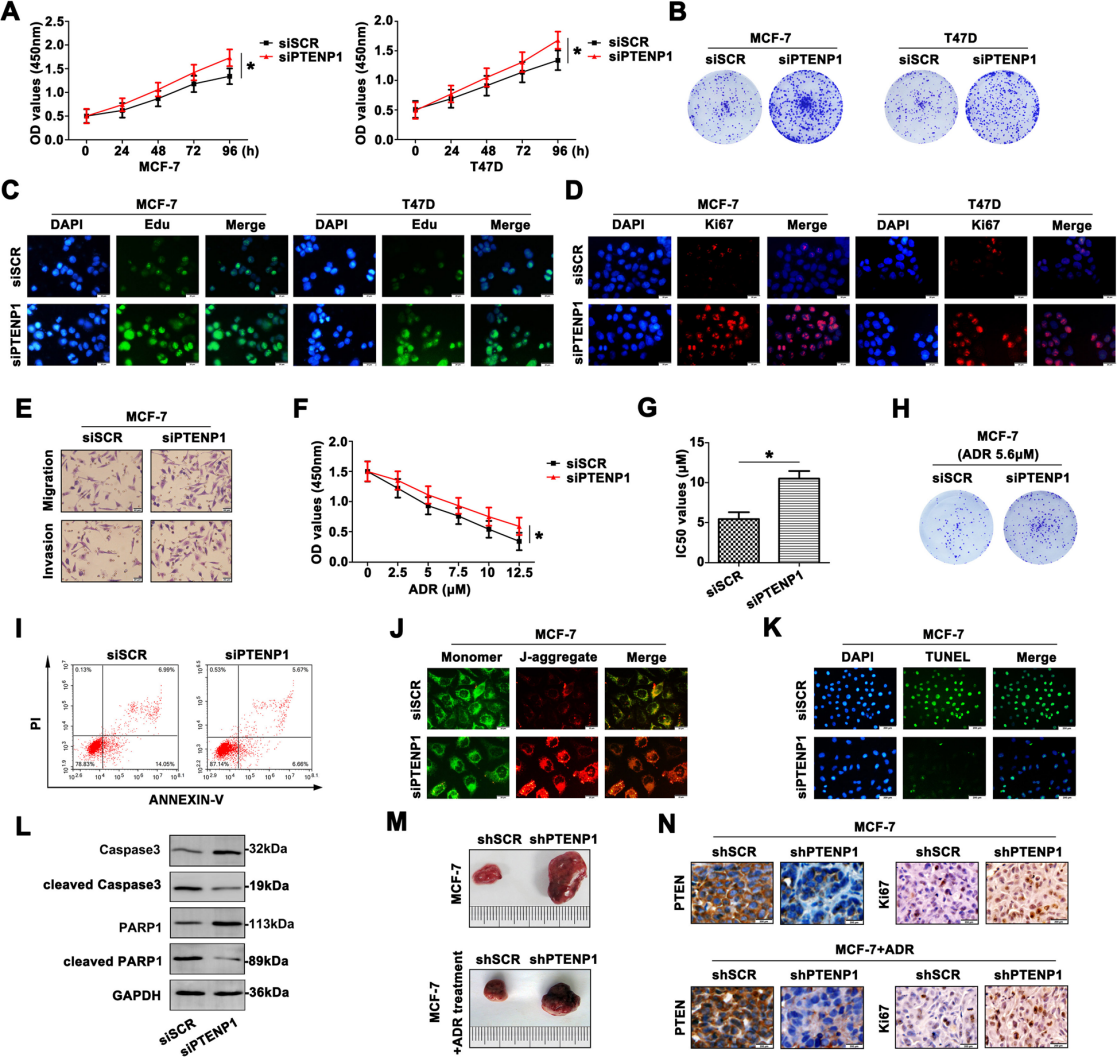
Low PTENP1 level enhances the malignant behavior of BC cells. **a** The viability of transfected BC cells were detected by CCK8 assays at 0, 24, 48,72, 96 h. **b** Knockdown of PTENP1 enhanced the colony formation in BC cells. **c** The proliferation of siPTENP1 transfected cells was increased by Edu staining (Scale bar = 20 μm). **d** Ki67 staining also showed intensive proliferation (Scale bar = 20 μm). **e** The aggressiveness was enhanced with knocking down PTENP1 in MCF-7 cells (Scale bar = 20 μm). **f** The siPTENP1-MCF-7 cells revealed more resistance to ADR. **g** Higher IC_50_ value was also proved the enhanced chemoresistance to ADR. **h** Weakened colony formation ability was shown in response to ADR. **i** More resistance to ADR was shown in siPTENP1-MCF-7 cells. Low apoptosis rate was detected by flow cytometry. **j** JC-1 staining assay showed altered mitochondrial membrane potential with siPTENP1 transfection. Green fluorescence: the monomer, red fluorescence: the J-aggregates, orange fluorescence: merged photo (Scale bar = 20 μm). **k** TUNEL assay confirmed the incidence of apoptosis (Scale bar = 200 μm). **l** Apoptosis-related molecules expression was determined by western blot. **m** The xenografted tumors were presented with or without ADR treatment. **n** PTEN and Ki67 levels were determined by IHC staining. Data are the means ± SD of triplicate determinants (*P < 0.05) (Scale bar = 200 μm)

**Fig. 6:**
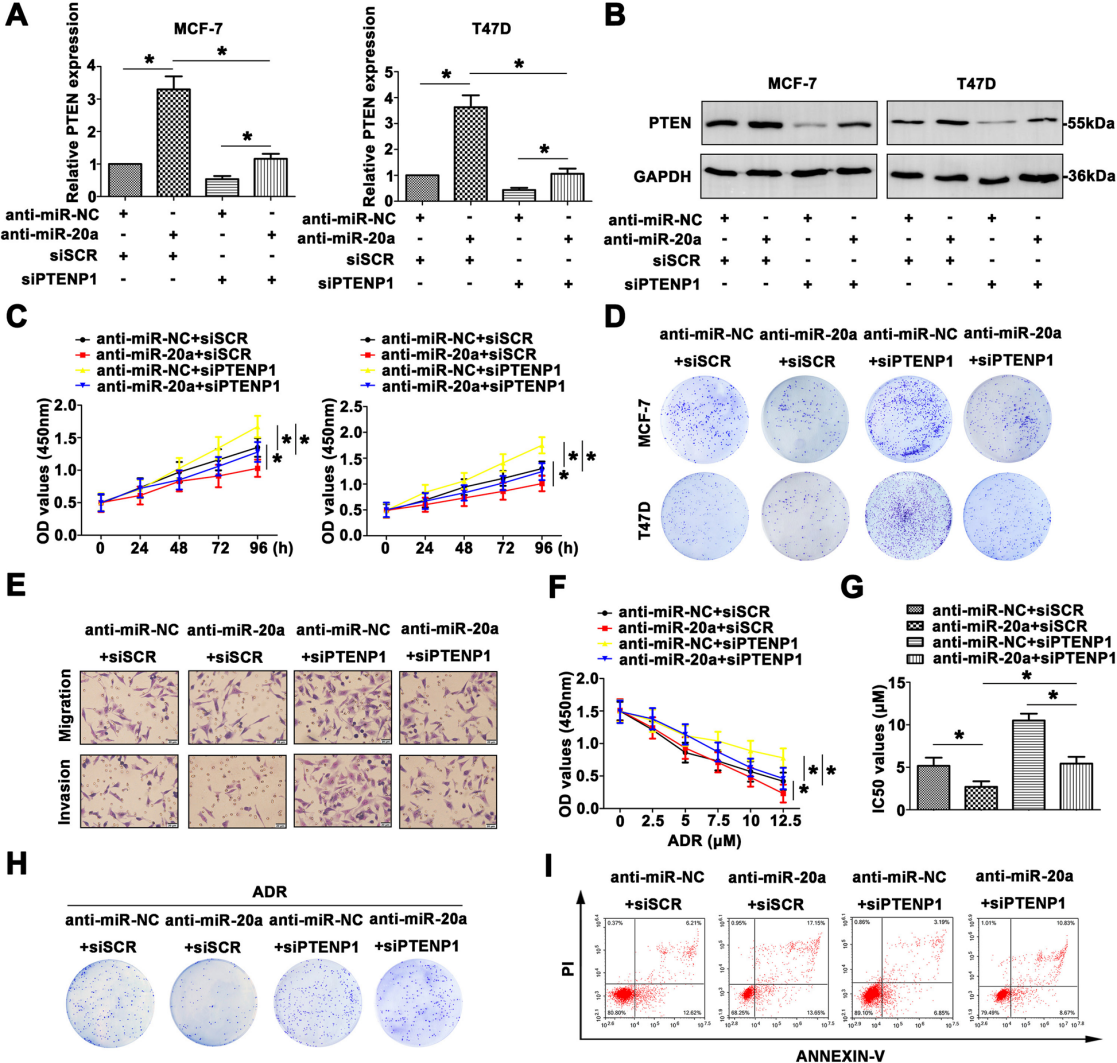
Inhibition of miR-20a reverses the promotional effect of siPTENP1 by mediating PTEN expression in BC progression. **a** PTEN mRNA expression was identified with the treatment of miR-20a inhibitor or siPTENP1. **b** PTEN protein level was detected by western blot. **c** The proliferation was measured by CCK8 assays. **d** Colony formation assay was used to measure the colony formation of transfected cells. **e** The aggressiveness was determined by transwell assay (Scale bar = 20 μm). **f** CCK8 assays were carried out to assess the chemoresistance to ADR with different treated BC cells. **g** IC_50_ values were calculated in differential treated MCF-7 cells. **h** In response to ADR, the colony formation was measured in transfected MCF-7 cells. **i** The AnnexinV and PI staining was used to determine the occurrence of apoptosis. Data are means ± SD of three independent assays (*P < 0.05)
