# Supplementary figures and images for: PTENP1/miR-20a/PTEN axis contributes to breast cancer progression by regulating PTEN via PI3K/AKT pathway
Source: J Exp Clin Cancer Res. 2019 Jun 13;38:256. doi: 10.1186/s13046-019-1260-6 (PMC6567415; doi:10.1186/s13046-019-1260-6)

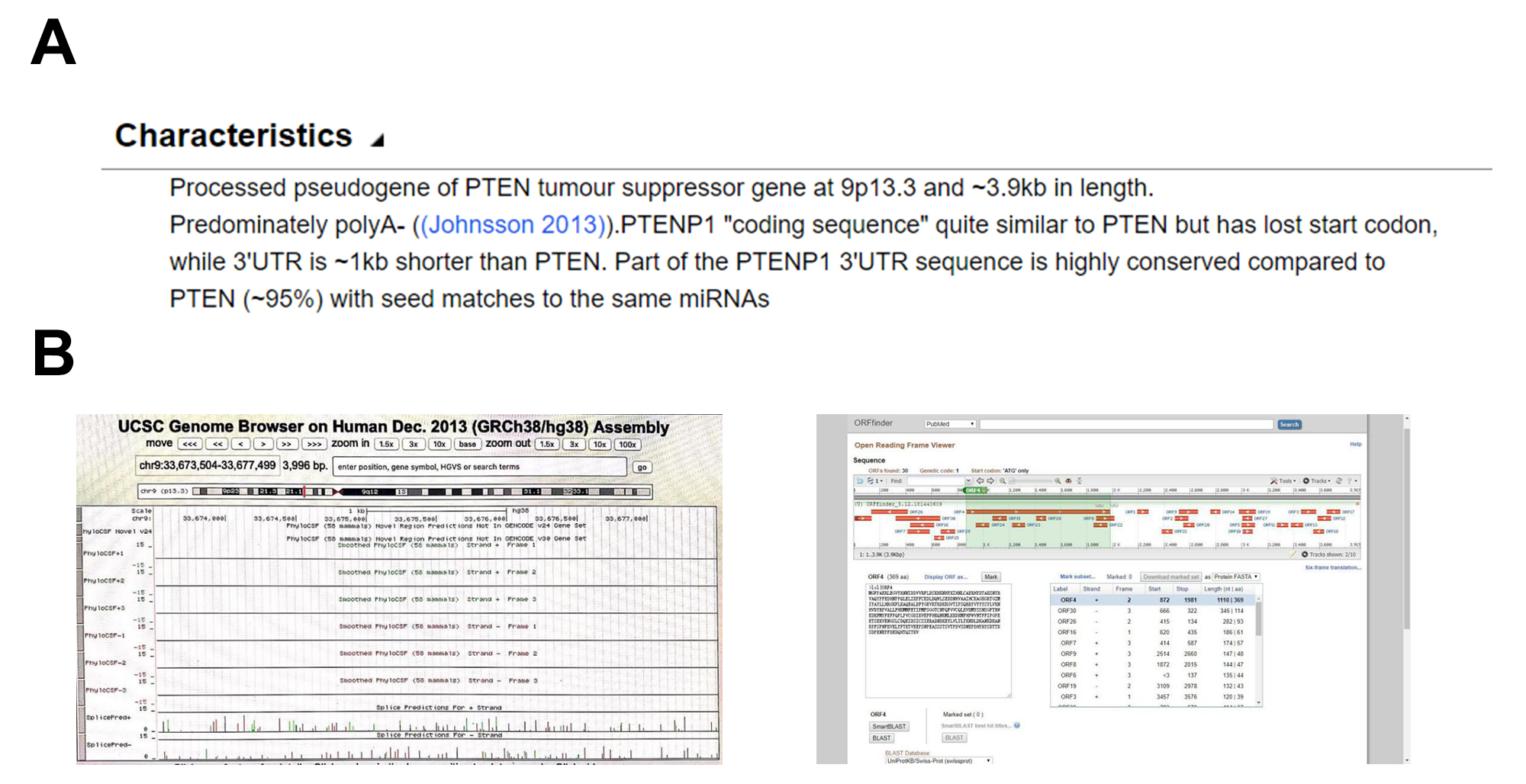

Supplement: Supplementary file 1 — Figure S1. Detailed information of PTENP1. (A) The predicted PTENP1 length was around 3.9kb by lncrnadb. (B) PTENP1 was predicted as non-coding RNA by ORF Finder and PhyloCSF analysis. (JPG 787 kb) [file 13046_2019_1260_MOESM1_ESM.jpg]

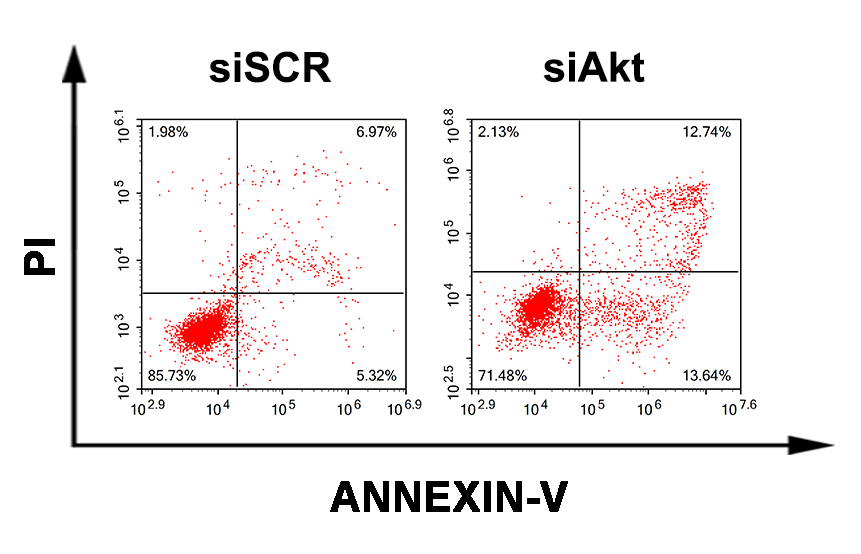

Supplement: Supplementary file 2 — Figure S2. SiAkt treatment induced highly apoptotic cell rates compared with the siSCR group. (JPG 265 kb) [file 13046_2019_1260_MOESM2_ESM.jpg]
